# Protein Arginine Methyltransferase 3 Enhances Chemoresistance in Pancreatic Cancer by Methylating hnRNPA1 to Increase ABCG2 Expression

**DOI:** 10.3390/cancers11010008

**Published:** 2018-12-20

**Authors:** Ming-Chuan Hsu, Mei-Ren Pan, Pei-Yi Chu, Ya-Li Tsai, Chia-Hua Tsai, Yan-Shen Shan, Li-Tzong Chen, Wen-Chun Hung

**Affiliations:** 1 National Institute of Cancer Research, National Health Research Institutes, Tainan 704, Taiwan; mchsu@nhri.org.tw (M.-C.H.); yali0147@gmail.com (Y.-L.T.); vanessatsai@nhri.org.tw (C.-H.T.); leochen@nhri.org.tw (L.-T.C.); 2 Graduate Institute of Clinical Medicine, College of Medicine, Kaohsiung Medical University, Kaohsiung 807, Taiwan; mrpan@cc.kmu.edu.tw; 3 Department of Pathology, Show Chwan Memorial Hospital, Changhua City 500, Taiwan; chu.peiyi@msa.hinet.net; 4 Department of Surgery, National Cheng Kung University Hospital, Tainan 704, Taiwan; ysshan@ncku.edu.tw; 5 Insitute of Clinical Medicine, College of Medicine, National Cheng Kung University, Tainan 704, Taiwan; 6 Department of Internal Medicine, National Cheng Kung University Hospital, Tainan 704, Taiwan; 7 Graduate Institute of Medicine, College of Medicine Kaohsiung Medical University, Kaohsiung 807, Taiwan

**Keywords:** PRMT3, hnRNP A1, ABCG2, RRM, drug resistance

## Abstract

Pancreatic cancer is poorly responsive to chemotherapy due to intrinsic or acquired resistance. Our previous study showed that epigenetic modifying enzymes including protein arginine methyltransferase 3 (PRMT3) are dysregulated in gemcitabine (GEM)-resistant pancreatic cancer cells. Here, we attempt to elucidate the role of PRMT3 in chemoresistance. Overexpression of PRMT3 led to increased resistance to GEM in pancreatic cancer cells, whereas reduction of PRMT3 restored GEM sensitivity in resistant cells. We identified a novel PRMT3 target, ATP-binding cassette subfamily G member 2 (ABCG2), which is known to play a critical role in drug resistance. PRMT3 overexpression upregulated *ABCG2* expression by increasing its mRNA stability. Mass spectrometric analysis identified hnRNPA1 as a PRMT3 interacting protein, and methylation of hnRNPA1 at R31 by PRMT3 in vivo and in vitro. The expression of methylation-deficient hnRNPA1-R31K mutant reduced the RNA binding activity of hnRNPA1 and the expression of *ABCG2* mRNA. Taken together, this provides the first evidence that PRMT3 methylates the RNA recognition motif (RRM) of hnRNPA1 and promotes the binding between hnRNPA1 and *ABCG2* to enhance drug resistance. Inhibition of PRMT3 could be a novel strategy for the treatment of GEM-resistant pancreatic cancer.

## 1. Introduction

Pancreatic cancer is a deadly disease with a 5-year survival rate of less than 8%. Although surgical resection is a potentially curative therapy for pancreatic cancer, only about 20% of patients whose primary tumors are localized are candidates for surgery [1]. For pancreatic cancer patients with locally advanced or metastatic tumors, chemotherapy is the standard treatment. Gemcitabine (GEM) has been used as a first-line treatment for pancreatic cancer patients since the late 1990s [2]. Pancreatic cancer patients initially respond to GEM but many patients later develop resistance after prolonged treatment. Currently, several mechanisms have been proposed to mediate the acquired GEM resistance in pancreatic cancer patients, including the reduced expression of nucleoside transporter [3,4], the alterations of nucleotide metabolism enzymes [5,6], and an increased drug efflux by ABC-transporters [7,8,9]. 

Emerging evidence suggests that epigenetic alterations, such as DNA methylation and histone modification, play an important role in regulating drug resistance [10,11]. For instance, DNA methylation of target of methylation-induced silencing-1 (TMS1), a pro-apoptotic gene, results in reduced TMS1 expression and increased GEM resistance. Inhibition of DNA methyltransferase by 5-azacytidine restores TMS1 expression and reactivates its apoptosis-inducing activity, thereby enhancing sensitivity of cancer cells to GEM [12]. GEM is a pro-drug that requires conversion into the active form after cellular uptake [13]. Promoter methylation of deoxycytidine kinase (dCK), which is critical for GEM metabolism, has been reported to repress mRNA expression and attenuate GEM sensitivity [14]. Moreover, dCK expression has been shown to correlate with GEM efficacy [5,15]. The involvement of histone modification enzymes in GEM resistance was recently reported in which depletion of enhancer zeste homolog 2 (EZH2), a histone methyltransferase responsible for the methylation of lysine 27 of histone H3 (H3K27), induces p27^Kip1^ expression and sensitizes pancreatic cancer cells to GEM [16]. 

Post-translational modification, such as protein methylation, is another mechanism that contributes to GEM resistance. For instance, protein arginine methyltransferase 1 (PRMT1), one of the nine members of the PRMT family which transfers methyl moieties from S-adenosylmethionine (SAM) to specific arginine residues on their substrates, methylates Gli1 to promote its oncogenic activity, resulting in reduced sensitivity of cells to GEM. Gli1 transcriptional factor, which is a key mediator in the Hedgehog signaling pathway, plays a critical role in pancreatic tumorigenesis. Depletion of PRMT1 effectively reversed GEM resistance in pancreatic cancer cells [17]. In addition, we previously demonstrated that euchromatic histone lysine methyltransferase 2 (EHMT2), a H3K9 methyltransferase, induces autocrine IL-8/CXCR1/2 stimulation in pancreatic cancer cells and paracrine activation of pancreatic stellate cells (PSCs) to increase GEM resistance. Inhibition of EHMT2 resensitizes pancreatic cancer cells to GEM in vitro, and the combination of EHMT2 inhibitor and GEM can overcome drug resistance in animal models [18]. In addition to EHMT2, we found that two other members of the PRMT family, PRMT3 and PRMT6, are highly upregulated in GEM-resistant pancreatic cancer cells [18]. The contribution of PRMT3 in chemoresistance is not well understood, and the effects of PRMT6 on the cytotoxicity of doxorubicin have only been reported in one study [19]. In the current study, we attempt to further our understanding of the molecular mechanisms by which PRMT3 and PRMT6 modulate the GEM resistance in pancreatic cancer. 

## 2. Results

### 2.1. PRMT3 Is Upregulated in GEM-Resistant Pancreatic Cancer Cells and Its Overexpression Increases the Resistance to Multiple Chemotherapeutic Drugs

**Figure 1:**
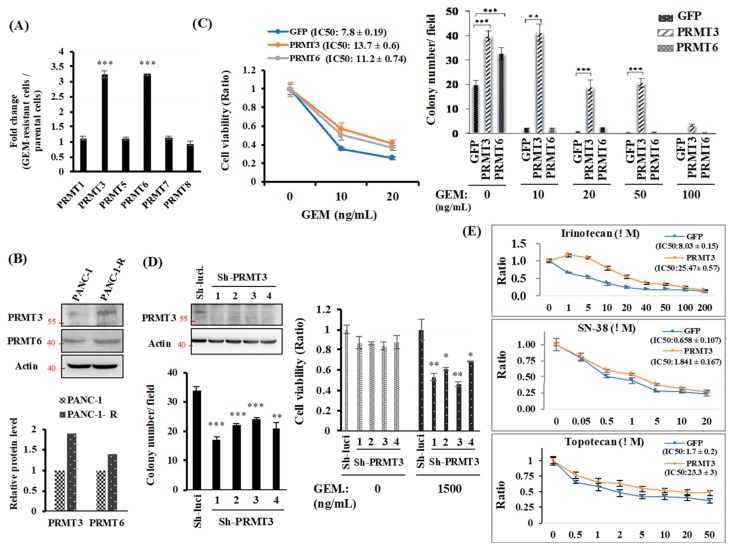
The expression of PRMT3 is upregulated in GEM-resistant pancreatic cancer cells and required for GEM-resistant activity. (**A**) Quantitative RT-PCR analysis of PRMTs expression. Fold change of PRMTs mRNA expression in PANC-1-R normalized to parental PANC-1 cells. *** *p* < 0.001. (**B**) The protein levels of PRMT3 and PRMT6 were detected by Western blot analysis. The intensity of the bands was quantified by Image J and normalized to that of actin. (**C**) Left: GFP-, PRMT3- and PRMT6-ovexpressing PANC-1 cells were treated with indicated concentrations of GEM for 5 days and then subjected to MTT assay to measure cell viability. Error bars, SEM. *n* = 3. IC50 values of GEM. in GFP-, PRMT3- and PRMT6-ovexpressing PANC-1 cells were presented as the mean ± standard deviation. Right: GFP-, PRMT3- and PRMT6-ovexpressing PANC-1 cells were subjected to anchorage-independent growth experiment. Quantitative analysis of colony numbers is shown. Error bars, SEM. *n* = 3. ** *p* < 0.01, *** *p* < 0.001. (**D**) Left: PRMT3-reduction PANC-1-R stable clones were subjected to anchorage-independent growth assay in culture medium containing 1500 ng/mL GEM for 2–3 weeks. The number of colonies was counted under microscope. Right: PRMT3-reduction PANC-1-R cells were incubated in the presence or absence of GEM and subjected to MTT assay. Error bars, SEM. *n* = 3. * *p* < 0.05, ** *p* < 0.01, *** *p* < 0.001. (**E**) GFP- and PRMT3-ovexpressing PANC-1 cells were treated with indicated concentrations of irinotecan, SN-38, and topotecan, respectively. After 3 days, cells were then subjected to MTT assay to measure cell viability. Error bars, SEM. *n* = 3. IC50 values of each compounds in GFP- and PRMT3-overexpressing PANC1 cells were presented as the mean ± standard deviation.

Results from our previous PCR array to compare the expression of epigenetic-modifying enzymes in PANC-1 and GEM-resistant PANC-1-R cells identified twenty-one enzymes that were upregulated in PANC-1-R cells [18]. Notably, gene expression of *PRMT3* and *PRMT6*, encoding protein arginine methyltransferases, was significantly upregulated in resistant cells compared with parental cells [20]. Given that little is known about the regulation of drug resistance by these two PRMTs, we sought to investigate the effects of upregulation of PRMT genes in pancreatic cancer cells. We first verified the expression of PRMTs by quantitative RT-PCR. Among the six PRMT genes examined, we observed substantially higher levels of *PRMT3* and *PRMT6* mRNA (Figure 1A) and protein (Figure 1B) in PANC-1-R cells compared with parental PANC-1 cells. To further establish the importance of PRMT3 and PRMT6 in GEM resistance, we generated PRMT3- and PRMT6-overexpressing stable clones from PANC-1 cells. Overexpression of PRMT3 and PRMT6 enhances cell growth (Supplementary Figure S1). Moreover, the results from cell viability assay showed that overexpression of PRMT3 and PRMT6 was more resistant to GEM compared to GFP-overexpressing PANC-1 cells (Figure 1C, left). Overexpression of PRMT3 or PRMT6 promoted anchorage-dependent cell growth. However, only PRMT3-overexpressing cells were resistant to GEM treatment (Figure 1C, right). Considering the inconsistence of PRMT6 overexpression in cell viability and anchorage-dependent cell growth, we only focus on PRMT3 and investigate its role in GEM resistance. As shown in Figure 1D, the reduction of PRMT3 resensitized PANC-1-R cells to GEM in both anchorage-dependent cell growth and cell viability assays. In addition to GEM, PRMT3-overexpressing cancer cells also showed increased resistance to irinotecan, SN-38, and topotecan, indicating that upregulation of PRMT3 contributes to multidrug resistance in pancreatic cancer cells (Figure 1E). 

### 2.2. PRMT3 Upregulates ABCG2 Expression

**Figure 2:**
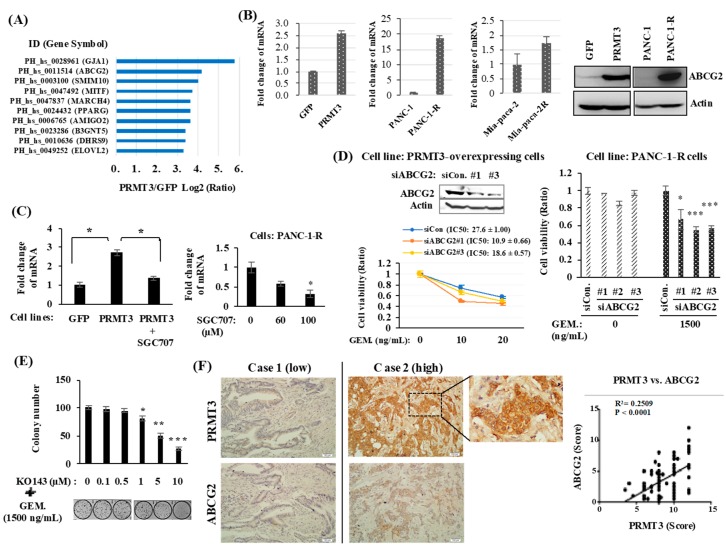
*ABCG2* is a target gene of PRMT3. (**A**) GFP- and GFP-PRMT3-overexpressing stable cells were used for gene expression microarray analysis to identify the potential target genes of PRMT3. Top ten increased genes are shown. (**B**) Total RNA and proteins were extracted from the indicated cell lines, and the levels of *ABCG2* mRNA and proteins were determined by quantitative RT-PCR and Western blot analysis, respectively. (**C**) PRMT3-overexpressng cells (left) and PANC-1-R cells (right) were treated with SGC707 and the level of *ABCG2* mRNA was determined by Quantitative RT-PCR analysis. * *p* < 0.05. (**D**) ABCG2 was depleted by siRNAs in PRMT3-overexpressing (left) and PANC-1-R (right) cells, respectively. These cells were cultured in the presence or absence of GEM for 5 days and then the cell viability was measured by MTT assay. Error bars, SEM. *n* = 3. * *p* < 0.05, *** *p* < 0.001. IC50 values of GEM. in PRMT3-ovexpressing PANC-1 cells with or without ABCG2 siRNA treatment were presented as the mean ± standard deviation. (**E**) PANC-1-R cells were treated with the combination of KO143 and GEM for 2 weeks. The number of colonies was counted and photographed. Error bars, SEM. *n* = 3. * *p* < 0.05, ** *p* < 0.01, *** *p* < 0.001. (**F**) Representative IHC staining of PRMT3 and ABCG2 in human PDAC tissues. A quantitative score for the protein expression of PRMT3 and ABCG2 was calculated from percentage of stained cells (X) multiplied by the immunostaining intensity (Y). The correlation between PRMT3 and ABCG2 in the 81 human tissue samples was determined by Pearson’s correlation coefficient.

We next investigated the mechanism underlying PRMT3-mediated GEM resistance. PRMT3 is a type I PRMT, which is known to induce asymmetric dimethylarginine (ADMA) methylation. Modification of histone H4R3 by PRMT3 is associated with gene transcription regulation. Moreover, PRMT3 might directly interact with the nuclear receptor liver X receptor α (LXRα) and acts as a transcription cofactor in methyltransferase-independent manner [21]. Given that PRMT3 might function as a transcriptional regulator, we studied the gene expression profiles in GFP- and PRMT3-overexpressing cells to identify potential genes that might be regulated by PRMT3 in GEM resistance. Of these top 10 genes that demonstrated increased expression (Figure 2A), the ATP binding cassette subfamily G member 2 (ABCG2), a member of the ABC transporter superfamily, which functions as a drug efflux pump, was significantly upregulated in PRMT3-overexpressing cells and has been reported to play a role in GEM resistance in pancreatic cancer [22,23,24]. Because the dysregulation of several genes, including hENT1, dCK, cytidine deaminase (CDA), and subunits of ribonucleotide reductase, has been proposed to modulate GEM resistance [25,26,27], we first assessed the expression of those genes in GEM-resistant cells. Our data showed that the expression of hENT1, CDA, dCK, and RRM2 was not altered in various GEM-resistant cell lines (Supplementary Figure S2). Further, we did not find the altered expression of those genes in our gene microarray data. Also, we did not find the increase of the members of ABCC subfamily. Only ABCG2 was significantly upregulated. Therefore, we focused on ABCG2 and validated its gene expression. As shown in Figure 2B, the expression of *ABCG2* mRNA was increased in PRMT3-overexpressing cells. In addition, *ABCG2* mRNA was upregulated in two GEM-resistant pancreatic cell lines, PANC-1-R and Mia-paca-2-R, when compared to the parental cells (Figure 2B). Increase of ABCG2 proteins was also confirmed in PRMT3-overexpressing cells and PANC-1-R cells (Figure 2B). Treatment of PRMT3-overexpressng cells or PANC-1-R cells with the PRMT3 inhibitor, SGC707, significantly reduced the expression of *ABCG2* (Figure 2C). To corroborate the importance of ABCG2 in GEM resistance, we first silenced ABCG2 by small interfering RNAs (siRNAs) in PRMT3-overexpressing cells and PANC-1-R cells and assayed for cell viability under GEM treatment. Loss of ABCG2 resulted in the increased sensitivity of cells to GEM (Figure 2D). Moreover, we treated PANC-1-R cells with the ABCG2 inhibitor KO143, which significantly enhanced the sensitivity of PANC-1-R cells to GEM (Figure 2E). The results suggested that the expression of ABCG2 is critical for mediating GEM resistance. The expression of PRMT3 and ABCG2 and their association were investigated in PDAC tumor tissues (*N* = 81) by immunohistochemical staining. Representative images of tumors with weak and strong staining of these two proteins are shown in Figure 2F. The results demonstrated a positive association between the expression of PRMT3 and ABCG2 in the tumors (*p* < 0.0001; Figure 2F, right). Together, these data suggested that upregulation of ABCG2 expression by PRMT3 contributes to GEM resistance in pancreatic cancer.

### 2.3. PRMT3 Upregulates the Expression of ABCG2 via Increased mRNA Stability

**Figure 3:**
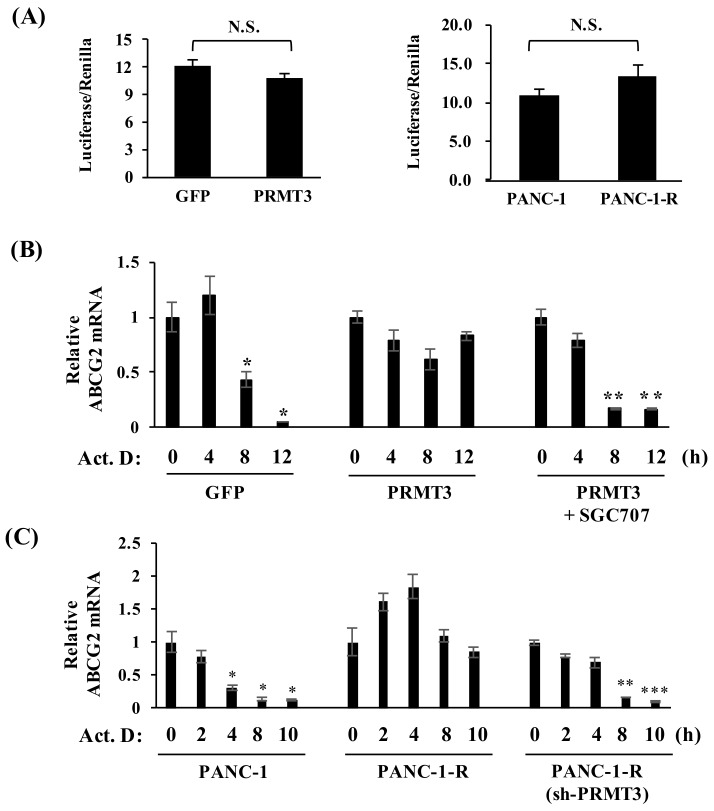
Overexpression of PRMT3 enhances the stability of *ABCG2* mRNA. (**A**) Luciferase activity of *ABCG2* promoter from the indicated cells was measured after transfection for 48 h. All values represent relative firefly luciferase activity normalized to the internal control. Error bars, SEM. *n* = 3. N.S.: not significant. (**B**) The indicated cells were treated with or without SGC707 in the presence of actinomycin D for the indicated time points and the levels of *ABCG2* mRNA were determined. All values represent relative mRNA level normalized to the control (0 h). Error bars, SEM. *n* = 3. * *p* < 0.05, ** *p* < 0.01. (**C**) The indicated cells were treated with actinomycin D for the indicated time points and the expression of *ABCG2* mRNA was measured. All values represent relative mRNA level normalized to control (0 h). Error bars, SEM. *n* = 3. * *p* < 0.05, ** *p* < 0.01, *** *p* < 0.001.

Next, we determined whether PRMT3 upregulates the expression of *ABCG2* via transcriptional activation. Unexpectedly, PRMT3 overexpression did not increase the promoter activity of the *ABCG2* gene in PANC-1 cells (Figure 3A, left). Similarly, the *ABCG2* promoter activity was not significantly altered in PANC-1-R cells (Figure 3A, right). These data suggested that PRMT3-mediated upregulation of *ABCG2* expression is independent of transcriptional activation. Those results led us to speculate that PRMT3 might enhance *ABCG2* expression by mediating post-transcriptional regulation. To this end, we treated GFP- and PRMT3-overexpressing cells with transcription inhibitor actinomycin D and evaluated *ABCG2* mRNA levels. Compared with GFP-overexpressing cells in which *ABCG2* mRNA levels decreased substantially after actinomycin D treatment for 12 h, overexpression of PRMT3 significantly prolonged *ABCG2* mRNA stability, which was reversed by the PRMT3 inhibitor (Figure 3B). Consistent with the above results, we observed a marked increase in the *ABCG2* mRNA stability in PANC-1-R cells, and that reduction of PRMT3 in PANC-1-R cells facilitated the degradation of *ABCG2* mRNA (Figure 3C). These results supported the notion that PRMT3 upregulates *ABCG2* expression by increasing its mRNA stability.

### 2.4. PRMT3 Interacts with hnRNP A1

**Figure 4:**
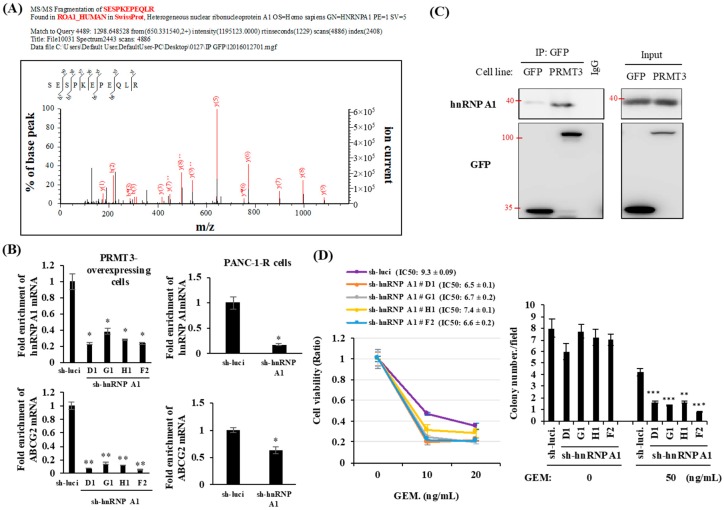
HnRNP A1 is a PRMT3 interacting protein. (**A**) GFP- and PRMT3-ovexpressing PANC-1 cells were subjected to immunoprecipitation using the GFP antibody followed by mass spectrometric analysis. (**B**) PRMT3-overexpressing cells (left panel) and PANC-1-R (right panel) were transfected with hnRNP A1-targeting shRNAs. mRNA levels of *hnRNP A1* and *ABCG2* were measured. Error bars, SEM. *n* = 3. * *p* < 0.05, ** *p* < 0.01. (**C**) Cell lysates were collected from GFP- and PRMT3-overexpressing cells and subjected to immunoprecipitation followed by Western blotting. (**D**) Left: PRMT3-overexpressing cells with or without hnRNP A1 reduction were cultured in the presence or absence of GEM for 5 days and then the cell viability was measured by MTT assay. Error bars, SEM. *n* = 3. IC50 values of GEM in PRMT3-ovexpressing PANC-1 cells with or without hnRNP A1 shRNA treatment were presented as the mean ± standard deviation. Right: PRMT3-overexpressing cells with or without hnRNP A1 reduction were subjected to soft agar assay. Cells were treated with GEM for 3 weeks and the number of colonies was counted under microscope. Error bars, SEM. *n* = 3. ** *p* < 0.01, *** *p* < 0.001.

To elucidate how PRMT3 increases the stability of *ABCG2* mRNA, we first searched the interacting proteins of PRMT3 to identify potential mediators involved in the regulation of mRNA stability. For this purpose, PRMT3-associated proteins were pulled down and identified by mass spectrometric analysis. A total of 293 potential PRMT3-associated proteins, including a well-known PRMT3 interaction partner, rpS2, were identified. We noticed that a number of heterogeneous nuclear ribonucleoproteins (hnRNPs), including hnRNP D and hnRNP A1, were potential PRMT3 interacting proteins (Supplementary Table S1). The hnRNP family modulates mRNA metabolism, splicing, and transcriptional and translational regulations [28,29]. To determine whether hnRNPs affect the expression of *ABCG2* mRNA, we depleted hnRNP D and hnRNP A1 by short hairpin RNAs (shRNAs). However, depletion of hnRNP D had no effects on *ABCG2* mRNA level (Supplementary Figure S3). We next studied hnRNP A1, another interacting partner of PRMT3 identified in our LC/MS/MS study (Figure 4A). Depletion of hnRNP A1 significantly reduced the expression of *ABCG2* mRNA in both PRMT3-overexpressing cells (Figure 4B, left panel) and PANC-1-R cells (Figure 4B, right panel). Thus, we focused on hnRNPA1 in all subsequent experiments. The interaction between PRMT3 and hnRNP A1 was further validated by co-immunoprecipitation assay (Figure 4C). To assess whether hnRNP A1 is involved in GEM resistance, hnRNP A1 was depleted by shRNAs in PRMT3-overexpressing cells. These cells were subjected to cell viability assay (Figure 4D, left) and anchorage-independent growth (Figure 4D, right) in the presence or absence of GEM. The results demonstrated that reduction of hnRNP A1 increased the sensitivity to GEM in these cells. Together, these data suggest that hnRNP A1 is a PRMT3-associated protein and is involved in the regulation of ABCG2 expression and GEM resistance.

### 2.5. PRMT3 Methylates hnRNP A1 at Arginine 31 (R31) In Vitro and In Vivo

**Figure 5:**
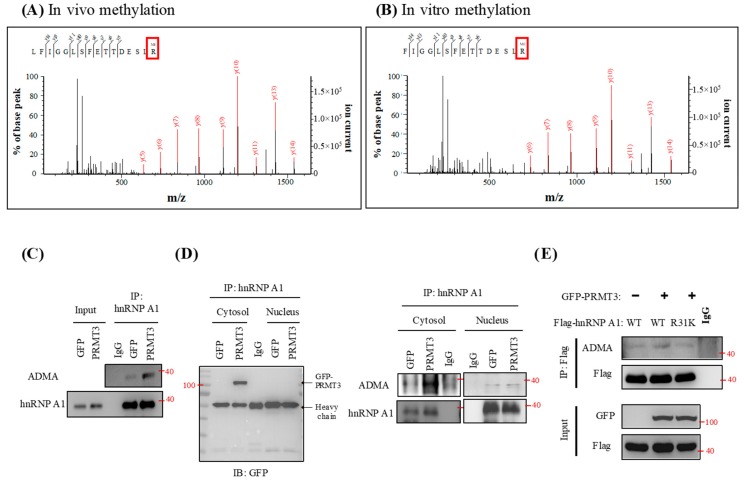
PRMT3 methylates hnRNP A1 at R31. (**A**) hnRNP A1 protein was purified from PRMT3-overexpressing cells and the immunoprecipitated complexes were separated by SDS-PAGE. The protein bands corresponding to hnRNP A1 were excised and subjected to mass spectrometric analysis. (**B**) Active human full-length PRMT3 and hnRNP A1 recombinant proteins were incubated together. The samples were separated by SDS-PAGE and the protein band corresponding to hnRNP A1 was excised and subjected to mass spectrometric analysis. (**C**) hnRNP A1 was immunoprecipitated from GFP- and GFP-PRMT3-overexpressing cells and subjected to Western blot. The level of ADMA and hnRNP A1 was detected. (**D**) hnRNP A1 was immunoprecipitated from both nuclear and cytoplasmic extracts of GFP- and GFP-PRMT3-overexpressing cells, followed by Western blotting with the indicated antibodies. (**E**) PANC-1 cells were co-transfected with GFP, GFP-PRMT3, Flag-tagged hnRNP A1-WT, or R31K mutant. Cell lysates were subjected to immunoprecipitation followed by Western blot analysis with the indicated antibodies.

To determine whether hnRNP A1 is a substrate of PRMT3, we purified hnRNP A1 protein from PRMT3-overexpressing cells. Mass spectrometric analysis of the purified hnRNP A1 indicated that the arginine residue at position 31 was methylated (Figure 5A). We also carried out in vitro methylation assay by using recombinant hnRNP A1 protein and identified a methylation signal at R31 from mass spectrometric analysis (Figure 5B). To further validate the methylation status of endogenous hnRNP A1, we immunoprecipitated hnRNP A1 and detected the asymmetric dimethylarginine (ADMA) status by Western blotting. We observed a marked increase in methylation of endogenous hnRNP A1 in PRMT3-overexpressing cells (Figure 5C). hnRNP A1 shuttles between the nucleus and cytoplasm [30], and PRMT3 is predominantly located in the cytoplasm [31]. Therefore, we speculated that PRMT3 interacts and methylates hnRNP A1 in the cytoplasm. To test this possibility, we performed subcellular fractionation and immunoprecipitated hnRNP A1 from both nuclear and cytoplasmic extracts. Our result confirmed that PRMT3 interacted with hnRNP A1 in the cytoplasm (Figure 5D, left panel). In addition, the major ADMA signal was detected in the cytoplasm (Figure 5D, right panel). These results are also consistent with the IHC data showing that PRMT3 mainly appeared in the cytoplasm (Figure 2F). Mutation of hnRNP A1 R31 abolished the increase of methylation of hnRNP A1 in PRMT3-overexpressing cells, suggesting that R31 of hnRNP A1 is the major methylation site for PRMT3 in vivo (Figure 5E). Together, these results indicated that hnRNP A1 is a bona fide substrate of PRMT3, which has not been previously reported. 

### 2.6. PRMT3-Mediated Methylation of hnRNP A1 Regulates the Binding of hnRNP A1 and ABCG2 mRNA

**Figure 6:**
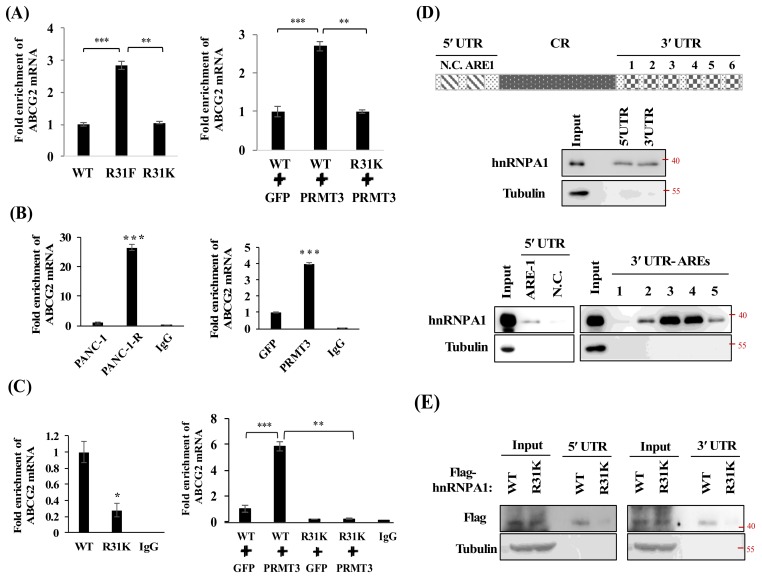
PRMT3-mediated R31 methylation of hnRNP A1 regulates the binding activity of hnRNP A1 toward ABCG2 mRNA. (**A**) Left: total RNA was extracted from hnRNP A1-WT-, R31F- and R31K-expressing cells and the levels of *ABCG2* mRNA were analyzed. Right: PANC-1 cells were cotransfected with indicated vectors. The levels of *ABCG2* mRNA were determined. Error bars, SEM. *n* = 3. ** *p* < 0.01, *** *p* < 0.001. (**B**) Total cellular lysates extracted from the indicated cells were incubated with anti-hnRNP A1 or mouse IgG antibodies, respectively. The hnRNP A1-associated RNAs were eluted and analyzed by real-time PCR for *ABCG2* mRNA levels. *** *p* < 0.001. (**C**) Left: total cellular lysates extracted from Flag-tagged hnRNP A1-WT, or R31K-expressing cells were incubated with anti-Flag or mouse IgG antibodies, respectively. Right: similarly, total cellular lysates extracted from PANC-1 cells which cotransfected with indicated vectors were incubated with anti-Flag or mouse IgG antibodies, respectively. The Flag-tagged hnRNP A1-associated RNAs were eluted and the levels of *ABCG2* mRNA were analyzed. Error bars, SEM. *n* = 3. * *p* < 0.05, ** *p* < 0.01, *** *p* < 0.001. (**D**) A schematic illustrating the AREs in *ABCG2* mRNA. One potential ARE in 5´UTR and six potential AREs in 3´UTR are shown. Individual ARE fragments were amplified and subjected to biotin RNA pull-down assay. The RNA-protein complexes were immunoprecipitated and analyzed by Western blot analysis using the hnRNP A1 and tubulin antibodies. (**E**) Flag-hnRNP A1-WT- and Flag-hnRNP A1-R31K-expressing PANC-1 cells were subjected to biotin RNA pull-down assay. The RNA-protein complexes were immunoprecipitated and analyzed by Western blot analysis using the Flag and tubulin antibodies.

To assess whether PRMT3-methylated R31 of hnRNP A1 affects the stability of *ABCG2* mRNA, we generated PANC-1 cells expressing hnRNP A1-WT, hnRNP A1-R31K, and hnRNP A1-R31F (methylation-mimicking mutant) and analyzed the *ABCG2* mRNA levels. As shown in Figure 6A, the hnRNP A1- R31F mutant demonstrated increased levels of *ABCG2* mRNA compared to hnRNP A1-WT- and hnRNP A1-R31K-expressing cells. Moreover, co-expression of PRMT3 and hnRNP A1-WT upregulated *ABCG2* mRNA level while co-expression of PRMT3 and hnRNP A1-R31K did not show any increase. These results suggested that PRMT3 upregulated the expression of *ABCG2* mRNA through R31 methylation of hnRNP A1. 

HnRNP A1 protein contains two RNA-recognition motifs (RRMs) in the N-terminal region, which play important roles in the regulation of RNA-binding specificity and affinity [32,33]. Because the R31 residue is located within the first RRM of hnRNP A1, we hypothesized that PRMT3-mediated R31 methylation might affect the RNA-binding affinity of hnRNP A1 to *ABCG2* mRNA. To test this possibility, we carried out RNA immunoprecipitation assay. As shown in Figure 6B, *ABCG2* mRNA was significantly enriched in PANC-1-R and PRMT3-overexpressing cells. Co-expression of PRMT3 and hnRNP A1-WT significantly increased the binding between hnRNP A1-WT and *ABCG2* mRNA, while co-expression of PRMT3 and hnRNP A1-R31K did not enhance the interaction (Figure 6C). To identify the binding regions of *ABCG2* mRNA for hnRNP A1, we amplified the 5′-UTR and 3′-UTR of *ABCG2* mRNA and labeled the RNA fragments for biotin RNA pull-down assay. Our results indicated that hnRNP A1 bound to both the 5′-UTR and 3′-UTR of *ABCG2* mRNA (Figure 6D, upper). Bioinformatics analysis revealed one potential AU-rich element (ARE), the AUUUA pentamer motif which is the potential site for hnRNP A1 binding [34,35], in the 5′-UTR and six potential AREs in 3′-UTR of *ABCG2* mRNA. hnRNP A1 bound to the 5’-UTR-ARE-1 but not to the negative control fragment (N.C.) that does not contain the ARE sequences (Figure 6D, bottom). In addition, hnRNP A1 bound strongly to the 3′-UTR-ARE-3 and 3′-UTR-ARE-4 sites, suggesting that these two AREs are the major binding sites for hnRNP A1 (Figure 6D, bottom). To further investigate whether PRMT3-mediated R31 methylation of hnRNP A1 regulates its binding to *ABCG2* mRNA, hnRNP A1-WT- and hnRNP A1-R31K-expressing PANC-1 cells were subjected to biotin RNA pull-down assay and results showed that hnRNP A1-R31K mutant reduced the binding activity to both the 5′-UTR and 3′-UTR of *ABCG2* mRNA (Figure 6E). These data suggest that PRMT3-mediated methylation of hnRNP A1 R31 enhances its binding to *ABCG2* mRNA.

## 3. Discussion

**Figure 7:**
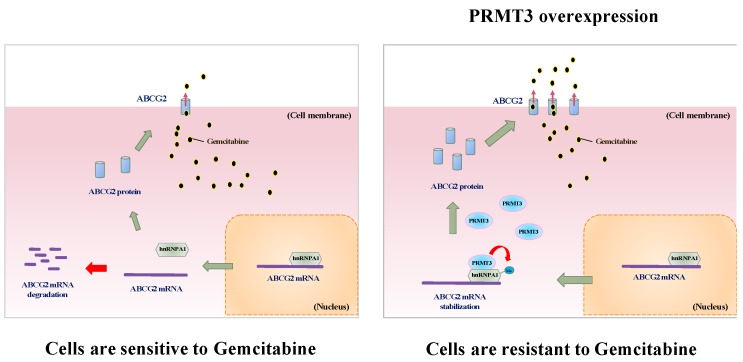
Proposed model of PRMT3-mediated GEM resistance in pancreatic cancer cells. PRMT3 is upregulated in GEM-resistant pancreatic cells and its overexpression stabilizes ABCG2 mRNA via methylating arginine 31 of hnRNPA1. PRMT3-mediated ABCG2 upregulation contributes to gemcitabine resistance.

PRMT3 belongs to type I PRMT family which attaches ADMA on its protein substrates. However, the biological functions of PRMT3 are still largely uncharacterized. The first physiological substrate of PRMT3 is the 40S ribosomal protein S2 [36], implying that PRMT3 might regulate ribosome biosynthesis and protein production. A potential role of PRMT3 in neuronal cells has been suggested by the findings that PRMT3 is highly expressed in different areas of the brain and is essential for the formation of dendritic spine in hippocampal neurons [37,38]. Recently, PRMT3 was reported to interact with LXRα to modulate hepatic lipogenesis and may participate in the onset of fatty liver [21]. The role of PRMT3 in tumorigenesis is not well characterized. Previously, studies have demonstrated that PRMT3 interacts with the tumor suppressor DAL-1/4.1B, which inhibits its methyltransferase activity in vitro and in vivo [39]. In this study, we provided the first evidence to demonstrate that PRMT3 plays an important role in GEM resistance (Figure 7, proposed model). Our results suggest that inhibition of PRMT3 might be a therapeutic strategy to improve the GEM sensitivity in pancreatic cancer treatment. Several selective and cell-permeable allosteric inhibitors of PRMT3 have been developed in the past several years [40,41]. It would be worthwhile to further evaluate the combination of PRMT3 inhibitors and GEM as a treatment option for pancreatic cancer in vivo.

By using an S-adenosylmethionine analogue as a probe, Guo et al. identified more than 80 potential PRMT3 substrates [42]. Whether these proteins are substrates of PRMT3 in vivo has not yet been validated; however, we found an interesting candidate, hnRNP A3, from the list. Comparing the amino acid sequences of human hnRNP A1, A2, and A3 (Supplementary Figure S4), the RRM1 motif is highly conserved in this hnRNP family. In addition, the R31 residue in hnRNP A1 is also highly conserved among the three members, suggesting that this residue may be an important site for methylation and functional regulation. To date, several arginine residues of hnRNP A1, including R190, R194, R233, R218, and R225, have been reported to be methylated by PRMT1 or PRMT5 in vitro and in vivo [43,44,45,46]. All of these arginine residues are located within the RGG domain in the C-terminal region. PRMT1 is a type I methyltransferase that catalyzes ADMA on arginine residues, whereas PRMT5 is a type II methyltransferase that is responsible for catalyzing symmetric dimethylarginine (SDMA). Thus, both ADMA and SDMA occur within the RGG motif of hnRNP A1. Two recent studies demonstrated that methylation of the RGG domain might regulate mRNA expression via modulation of the internal ribosome entry site (IRES)-dependent translation [43,44]. We identified a distinct region, the RRM domain, of hnRNP A1, which can be modified via ADMA by PRMT3, and this methylation enhances the binding of hnRNP A1 to target mRNAs. Unlike the RGG domain, the functional role of arginine methylation within the *N*-terminal RRM domains of RNA-binding proteins has not been explored. A previous study revealed that PRMT4 methylates the RNA-binding protein HuR at one arginine residue located within the hinge region between two RRM motifs, which results in the induction of TNF-α expression via the stabilization of mRNA [47]. Our results supported the hypothesis that arginine methylation within the RRM domains of hnRNP A1 promotes its RNA-binding activity and increases the stability of its target mRNA. The RRM motifs of hnRNP A1 have also been reported to bind to telomeric DNA and be involved in telomere length maintenance [48]. It is possible that PRMT3-mediated methylation in the RRM domain of hnRNP A1 might play a role in regulating the binding of hnRNP A1 to telomeric DNA and the maintenance of telomere length.

It is well known that hnRNP A1 binds to the pre-mRNAs during the splicing process in the nucleus and might assist in the delivery of mature mRNAs to the cytoplasm. After its release from the mRNA for protein translation in the cytoplasm, hnRNP A1 interacts with nuclear import protein to be translocated back into the nucleus [30,49,50]. Because PRMT3 is highly abundant in the cytoplasm, methylation of hnRNP A1 by PRMT3 might occur primarily in the cytoplasm. So far, because there is no evidence to indicate the involvement of RRM domain of hnRNP A1 in nuclear-cytoplasmic shuttling, whether PRMT3 affects the subcellular localization of hnRNP A1 through arginine methylation of RRM domain remains unclear. In addition, it is possible that methylation of hnRNP A1 by PRMT3 increases its binding to the target mRNAs, which induces the accumulation of methylated hnRNP A1 in the cytoplasm. However, we could not exclude the possibility that PRMT3 methylates hnRNP A1 in the nucleus and the methylated hnRNP A1 forms a stable complex with target mRNAs, which is continuously exported to the cytoplasm. Therefore, the methylated hnRNP A1 is preferentially detected in the cytoplasm. 

The role of ABCG2 in gemcitabine resistance is still controversial. Several studies do not support the involvement of ABCG2 in gemcitabine resistance [27,51]. However, some studies indeed demonstrated that ABCG2 contributes to gemcitabine resistance [22,23,52,53,54]. In our microarray data, we did not find the change of hENT1, hCNT1, dCK, and subunits of ribonucleotide reductase in the PRMT3-overexpressing PANC-1 cells. Only ABCG2 was significantly upregulated. Therefore, our results suggested that ABCG2 is one of the key mediators of gemcitabine resistance.

## 4. Material and Methods

### 4.1. Cell Culture and Reagents

Pancreatic cancer cell lines PANC-1 and Mia-Paca-2 were purchased from ATCC and had been re-authenticated by short tandem repeat profiling in 2014. Cells were cultured in DMEM medium with 10% fetal bovine serum (FBS) and 1% penicillin/streptomycin. The GEM-resistant cell line, PANC-1-R, was established from parental human PANC-1 pancreatic cancer cells as previously described [20]. PANC-1 cells with stable expressions of GFP, GFP-PRMT3, and GFP-PRMT6, were grown in the DMEM medium supplemented with 800 μg/mL G418. PANC-1-R cells with PRMT3 reduction were maintained in DMEM medium containing 1500 ng/mL of GEM and 1 μg/mL puromycin. GFP-PRMT3 stable cells with hnRNP A1 reduction were maintained in DMEM medium containing 800 μg/mL of G418 and 1 μg/mL puromycin. All cell lines were authenticated with short tandem repeat profiling. The following antibodies were used in this study: PRMT3 (Cat No. GTX23765, GeneTex, Irvine, CA, USA); PRMT6 (Cat No. A300-929A, Bethyl Laboratories, Montgomery, TX, USA); ABCG2/BCRP (Cat No. MAB4146, Millipore, Billerica, MA, USA); hnRNP A1 (Cat No. 05-1521, Millipore); GFP (Cat No. ab290, Abcam, Cambridge, UK); asymmetrical dimethyl arginine (ADMA) (Cat No. 13522, Cell Signaling Technology, Danvers, MA, USA); and Flag M2 (Cat No. F1804, Sigma, St. Louis, MO, USA). GEM (Gemzar, Lilly, Indianapolis, IN, USA) stocks were stored in aliquots at 4 °C. Irinotecan (Cat No. I1406) was obtained from Sigma. SN-38 (CAS No. 15632), Topotecan (CAS No. 14129), and PRMT3 inhibitor SGC707 (CAS No. 1687736-54-4) were purchased from Cayman Chemical (Ann Arbor, MI, USA). ABCG2 inhibitor KO143 (Cat No. SC-204030) was ordered from Santa Cruz Biotechnology (Santa Cruz, CA, USA). 

### 4.2. Plasmids

The pEGFP-PRMT3 and -PRMT6 plasmids were kindly provided by Dr. Mien-Chie Hung [55]. Human hnRNP A1 cDNA ORF Clone (Cat No. NM-031157) was purchased from OriGene (Rockville, MD, USA). ABCG2 promoter (pGL4-BCRP) was kindly provided by Dr. Wei-Chien Huang [56]. PRMT3 shRNA and hnRNP A1 shRNA plasmids were purchased from National RNAi Core, Taiwan. The sequences of shRNA plasmids are shown in Supplementary Table S2. 

### 4.3. Soft Agar Colony Formation Assay

To prepare the bottom layer of agar, 0.6% agarose containing DMEM medium was added into the six-well plate and set aside for 10 min to allow agarose to solidify. Cells suspended in DMEM medium containing 0.3% agarose were plated onto the bottom layer of agar at a density of 2000 cells per well. After incubation for 2–3 weeks, colonies larger than 50 µm in diameter were counted under microscopy. The results were expressed as the means ± SE of three independent experiments.

### 4.4. Real-Time Reverse Transcription Polymerase Chain Reaction (RT-PCR) 

Total RNA was extracted by using RNeasy mini kit and then reverse-transcribed into cDNA using the RT2 first strand kit (Qiagen, Hilden, Germany). The PCR experiments were done by 7500 Fast Real-time PCR system (Applied Biosystems, Foster City, CA, USA). The sequences of primers used for real-time PCR analysis are shown in Supplementary Table S2.

### 4.5. Site-Directed Mutagenesis

Flag-tagged hnRNP A1-R31K and -R31F mutants were generated using QuickChange Site-Directed Mutagenesis Kit according to the manufacturer’s protocol (Stratagene, La Jolla, CA, USA). The primers used for mutagenesis are shown in Supplementary Table S2.

### 4.6. Mass Spectrometry

GFP-PRMT3 and hnRNP A1 were purified from GFP-PRMT3-overexpressing cells by immunoprecipitation with GFP and hnRNP A1 antibodies, respectively. The GFP-purified complexes were subjected to in-solution digestion with trypsin and the PRMT3-interacting proteins were identified by mass spectrometry (Mithra Biotechnology Inc., New Taipei City, Taiwan). To identify the arginine residue on hnRNP A1 methylated by PRMT3, the hnRNP A1-purified complexes were separated by SDS-PAGE. The protein bands corresponding to hnRNP A1 were excised and subjected to in-gel digestion with trypsin, and the methylated arginine residue was analyzed by liquid chromatography/tandem mass spectrometry (Mithra Biotechnology Inc., New Taipei City, Taiwan). 

### 4.7. In Vitro Methylation Assay

Active recombinant full-length human PRMT3 protein (Cat No. ab167953, Abcam) was purchased from Abcam. Human hnRNP A1 recombinant protein (Cat No. pro-1038) was purchased from PROSPEC. PRMT3 (1 μg) was incubated with hnRNP A1 (2 μg) in the presence of 40 μM S-adenosyl-L-methionine (Sigma) for 1 h at 30 °C in a final volume of 25 μL of phosphate-buffered saline. After incubation, sample was separated by SDS-PAGE, and the protein band corresponding to hnRNP A1 was excised for mass spectrometry. 

### 4.8. RNA Immunoprecipitation (RIP) Assay

RIP assay was performed according to the manufacturer’s protocol (Cat No. 03-204, Millipore). In brief, cells were lysed with RIP lysis buffer, and cell extracts were incubated with 5 μg of normal mouse IgG or hnRNP A1 antibodies at 4 °C overnight. After incubation, 50 μL of magnetic beads were added to the immunoprecipitated complexes. The beads were washed six times with RIP wash buffer, followed by protein digestion in proteinase K buffer at 55 °C for 30 min. RNA was purified by phenol-chloroform extraction and subjected to real-time RT-PCR.

### 4.9. Biotinylated RNA Pull-Down Assay

Complementary DNA prepared from the mRNA of PANC-1 cells was used as a template for PCR amplification of different regions of ABCG2 mRNA. All forward primers contained the T7 RNA polymerase promoter sequence 5′-CCAAGCTTCTAATACGACTCACTATAGGGAGA-3′ (T7). All of the primer pairs were used to prepare the templates, including the 5′UTR (1-493), 3′UTR (2462-4445), 5′UTR-N.C. (1-295), 5′UTR-ARE1 (271-550), 3′UTR-ARE1 (2381-2573), 3′UTR-ARE2 (2553-2821), 3′UTR-ARE3 (2801-3062), 3′UTR-ARE4 (3276-3585), and 3′UTR-ARE5 (4027-4331) of ABCG2 mRNA. The sequences of primers are shown in Supplementary Table S2. Following PCR amplification, PCR fragments containing the T7 RNA polymerase promoter sequence were purified by gel electrophoresis. To prepare biotinylated RNA probes, the purified PCR fragments were incubated with T7 RNA polymerases (Cat No. P2075, Promega, Madison, WI, USA) and biotin RNA-labeling mixture (Cat No. 11685597910, Sigma) for 2 h at 37 °C, followed by DNase I treatment for 15 min at 30 °C to remove template DNA. The biotinylated RNA was purified by phenol-chloroform extraction and rehydrated in DEPC-treated water. Purified biotinylated RNA (500 ng) was incubated with 500 μg of cell lysates overnight at 4 °C, and RNA/proteins complexes were immunoprecipitated using the streptavidin-conjugated Dynabeads and subjected to SDS-PAGE followed by Western blot analysis using hnRNP A1 and tubulin antibodies.

### 4.10. Immunohistochemical (IHC) Staining and Analysis

Human PDAC tissues were obtained from patients undergoing surgical resection in National Cheng Kung University Hospital (Tainan, Taiwan) under the guidelines approved by the Institutional Review Board of National Cheng Kung University Hospital (Protocal No/IRB No: /B-ER-106-230). Written informed consent was obtained from each patient [57]. Tissue sections were stained with human PRMT3 (Cat No. GTX23765, GeneTex) and human ABCG2 (Cat No. MAB4146, Millipore) antibodies overnight at 4 °C followed by incubation with horseradish peroxidase (HRP)-conjugated secondary antibodies for 1 h at room temperature. The protein signal was developed using a 3,3′-diaminobenzidine solution, and sections were evaluated under microscope by a pathologist. The staining intensity and average percentage of positive cells were analyzed using ten independent high-magnification fields. The intensity of the staining was classified into four grades: 0, negative staining; 1, weak staining; 2, moderate staining; 3, strong staining. The percentage of positive cells was categorized into the following grades: 0, <10%; 1, 10–25%; 2, 25–50%; 3, 50–75%; 4, >75%. The results (0–12) were scored by multiplying the staining intensity and the percentage of positive cells.

### 4.11. Statistics Analyses

The two-tailed unpaired *t*-test was used to assess the difference between two independent experimental groups and *p*-value < 0.05 was considered statistically significant. The correlation between PRMT3 and ABCG2 expression in human tissue samples was determined by Pearson’s correlation coefficient.

## 5. Conclusions

In summary, we identify hnRNP A1 as a novel substrate of PRMT3, and PRMT3-methylated hnRNP A1 at R31 enhances its binding to the ABCG2 mRNA, leading to upregulation of ABCG2 and resistance to GEM. Therefore, targeting PRMT3 may be a potential therapeutic approach for the treatment of GEM-resistant pancreatic cancer. (Figure 7, proposed model).

## Supplementary Materials

The following are available online at http://www.mdpi.com/2072-6694/11/1/8/s1, Figure S1: Overexpression of PRMT3 and PRMT6 facilitates cell proliferation, Figure S2: The expression of hENT1, RRM2, CDA and dCK was not altered in gemcitabine-resistant cell lines, Figure S3: Depletion of hnRNP D had no effects on ABCG2 mRNA level, Figure S4: Sequence alignment of hnRNP A1, A2 and A3 proteins, Table S1: PRMT3-associated heterogeneous nuclear ribonucleoproteins (hnRNPs), Table S2: Oligonucleotide sequences.
