# Supplementary material for: Protein Arginine Methyltransferase 3 Enhances Chemoresistance in Pancreatic Cancer by Methylating hnRNPA1 to Increase ABCG2 Expression
Source: Cancers (Basel). 2018 Dec 20;11(1):8. doi: 10.3390/cancers11010008 (PMC6356582; doi:10.3390/cancers11010008)
Supplement: Supplementary file 1 [file cancers-11-00008-s001.pdf]

Supplementary Materials

Protein Arginine Methyltransferase 3 Enhances Chemoresistance in Pancreatic Cancer by Methylating hnRNPA1 to Increase ABCG2 Expression

Ming-Chuan Hsu, Mei-Ren Pan, Pei-Yi Chu, Ya-Li Tsai, Chia-Hua Tsai, Yan-Shen Shan, Li-Tzong Chen and Wen-Chun Hung

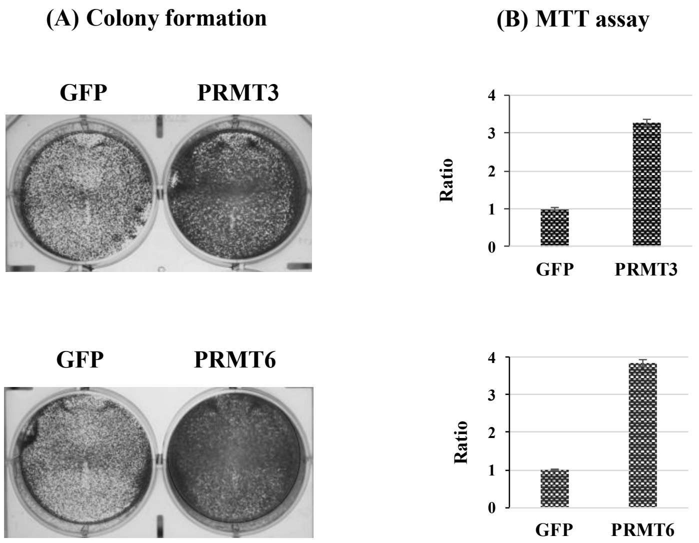

Figure S1. Overexpression of PRMT3 and PRMT6 facilitates cell proliferation. (**A**) GFP-, PRMT3- and PRMT6-overexpressing PANC-1 cells (1×10^4) were cultured for three days and the number of colonies was photographed. (**B**) GFP-, PRMT3- and PRMT6-overexpressing PANC-1 cells were cultured for three days and then the cell viability was measured by MTT assay. Error bars, SEM. n = 3.

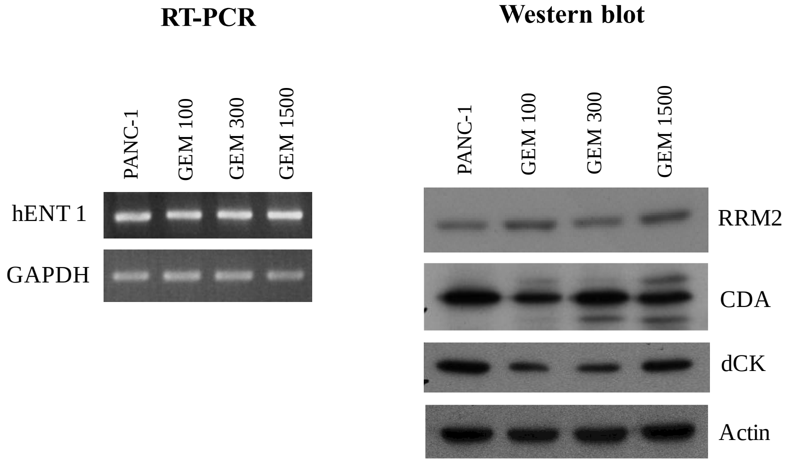

Figure S2. The expression of hENT1, RRM2, CDA and dCK was not altered in gemcitabine-resistant cell lines.

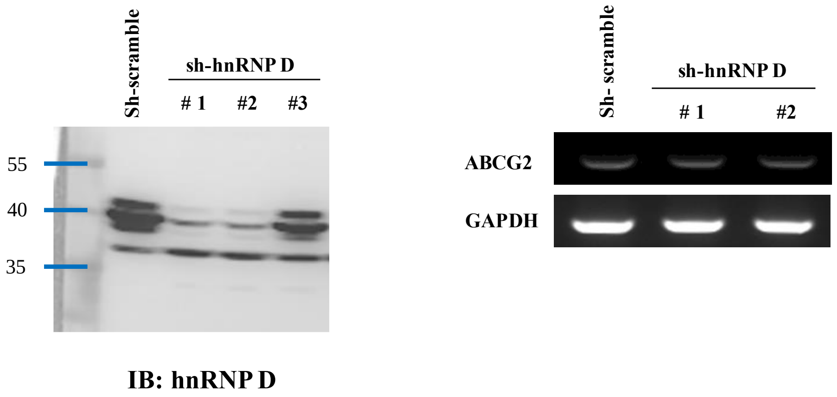

Figure S3. Depletion of hnRNP D had no effects on ABCG2 mRNA level. PRMT3-overexpressing PANC-1 cells were transfected with hnRNP D-targeting shRNAs. The protein level of hnRNP D was determined by western blot and mRNA levels of ABCG2 was measured by semi-quantitative RT-PCR.

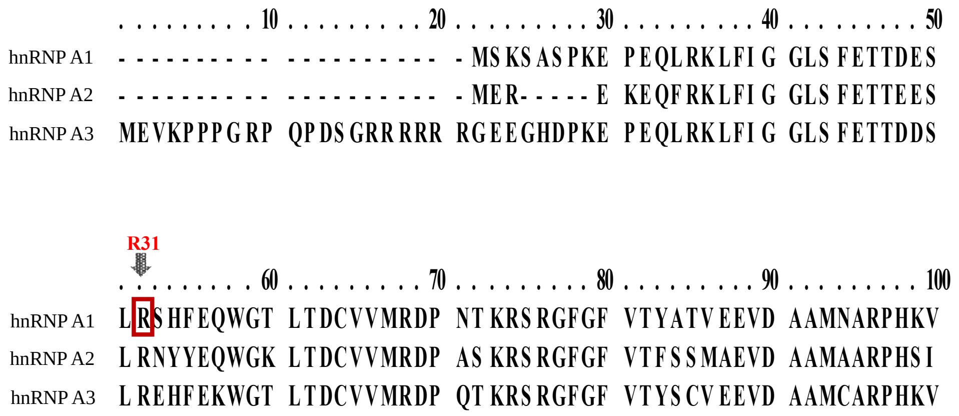

Figure S4. Sequence alignment of hnRNP A1, A2 and A3 proteins.

Table S1. PRMT3-associated heterogeneous nuclear ribonucleoproteins (hnRNPs).

HNRPK_HUMAN	Heterogeneous nuclear ribonucleoprotein K OS = Homo sapiens GN = HNRNPK PE = 1 SV = 1
HNRPD_HUMAN	Heterogeneous nuclear ribonucleoprotein D0 OS = Homo sapiens GN = HNRNPD PE = 1 SV = 1
HNRH1_HUMAN	Heterogeneous nuclear ribonucleoprotein H OS = Homo sapiens GN = HNRNPH1 PE = 1 SV = 4
ROA1_HUMAN	Heterogeneous nuclear ribonucleoprotein A1 OS = Homo sapiens GN = HNRNPA1 PE = 1 SV = 5
HNRPF_HUMAN	Heterogeneous nuclear ribonucleoprotein F OS = Homo sapiens GN = HNRNPF PE = 1 SV = 3
HNRDL_HUMAN	Heterogeneous nuclear ribonucleoprotein D-like OS = Homo sapiens GN = HNRNPDL PE = 1 SV = 3
ROA3_HUMAN	Heterogeneous nuclear ribonucleoprotein A3 OS = Homo sapiens GN = HNRNPA3 PE = 1 SV = 2

Table S2. Oligonucleotide sequences.

**The Sequences of shRNA Plasmids**	
sh-PRMT3#1	CGTGACCCTCACGTTGAATAA
sh-PRMT3#2	CCTTGGGAGAAAGAAGAGAT
sh-PRMT3#3	CCTTGTGGTATTAAGCATATA
sh-PRMT3#4	GCAGTGAGTGATGTGAATAAA
sh-hnRNP A1#D1	AGATATTTGTTGGTGGCATTA
sh-hnRNP A1#G1	ACCCATGAAGGGAGGAAATTT
sh-hnRNP A1#H1	CAACAATCAGTCTTCAAATTT
sh-hnRNP A1#F2	AGATATTTGTTGGTGGCATTA
**The Sequences of Primers (5′–3′)**	
PRMT3-F	GCATGGTTCATAAACATGGACTT
PRMT3-R	CACCCAACATCCAAAACTACC
PRMT6-F	CCTGGGTATCCTTCGGAACT
PRMT6-R	CTCCTTCAGCCACTTGGTTC
ABCG2-F	ACTGAGATTGAGAGACGCGG
ABCG2-R	TCTGGAGAGTTTTTATCTTTTCAGC
hnRNP A1-F	CCACGAAACCAAGGTGGCTA
hnRNP A1-R	CTGGCTCTCCTCTCCTGCTA
hnRNPA1-R31K-F	CAACTGATGAGAGCCTGAAGAGCCATTTTGAGCAATGG
hnRNPA1-R31K-R	CCATTGCTCAAAATGGCTCTTCAGGCTCTCATCAGTTG
hnRNPA1-R31F-F	CAACTGATGAGAGCCTGTTTAGCCATTTTGAGCAATGG
hnRNPA1-R31F-R	CCATTGCTCAAAATGGCTAAACAGGCTCTCATCAGTTG
5′UTR (1-493)-F	(T7) GTCAGCGCTGCCTGAGCTCG
5′UTR (1-493)-R	GCCATTGGTGTTTCCTTGTGACACTGGG
3′UTR (2462-4445)-F	(T7) CACGTGGCCTTGGCTTGTATG
3′UTR (2462-4445)-R	CTCTCTGTGTAATAAGGGAAGGG
5′UTR-N.C. (1-295)-F	(T7) GTCAGCGCTGCCTGAGCTCG
5′UTR-N.C. (1-295)-R	CCTAAATCCTACCCAGTTCCTCCAC
5′UTR-ARE1 (271-550)-F	(T7) GTGGAGGAACTGGGTAGGATTTAGG
5′UTR-ARE1 (271-550)-R	GCCATTGGTGTTTCCTTGTGACACTGGG
3′UTR-ARE1 (2381-2573)-F	(T7) CACGTGGCCTTGGCTTGTATG
3′UTR-ARE1 (2381-2573)-R	GCAACAGTGTGATGGCAAGGG
3′UTR-ARE2 (2553-2821)-F	(T7) CCCTTGCCATCACACTGTTGC
3′UTR-ARE2 (2553-2821)-R	GGGCTACTAACCTACCTATTC
3′UTR-ARE3 (2801-3062)-F	(T7) GAATAGGTAGGTTAGTAGCCC
3′UTR-ARE3 (2801-3062)-R	CCAAACCCTCAGCCAAGATTCC
3′UTR-ARE4 (3276-3585)-F	(T7) CTGACCACTCCTGGATTTGG
3′UTR-ARE4 (3276-3585)-R	GGAAGGAAGTAGTGACTGGGAG
3′UTR-ARE5 (4027-4331)-F	(T7) GGGGCATCATGGTGTATAGACGCC
3′UTR-ARE5 (4027-4331)-R	CTCTCTGTGTAATAAGGGAAGGG

© 2018 by the authors. Licensee MDPI, Basel, Switzerland. This article is an open access article distributed under the terms and conditions of the Creative Commons Attribution (CC BY) license (http://creativecommons.org/licenses/by/4.0/).
